# Onchocerca ochengi male worms implanted in SCID mice and Gerbil: Relationship between microfilaridermia status of cows, nodular worm viability and fertility and worm survival in the rodents

**DOI:** 10.1016/j.exppara.2021.108143

**Published:** 2021-10

**Authors:** Desmond N. Akumtoh, Abdel J. Njouendou, Haelly M. Metuge, Hanna T. Sjoberg, Nicolas P. Pionnier, Valerine C. Chunda, Narcisse Victor T. Gandjui, Lontum B. Ndzeshang, Fanny F. Fombad, Raphael A. Abong, Peter A. Enyong, Jerome Fru-Cho, Mathias E. Esum, Manuel Ritter, Mark J. Taylor, Joseph D. Turner, Samuel Wanji

**Affiliations:** a Research Foundation for Tropical Diseases and the Environment (REFOTDE), Buea, Cameroon; b Parasite and Vector Research Unit (PAVRU), Department of Microbiology and Parasitology, University of Buea, Buea, Cameroon; c Department of Biomedical Sciences, Faculty of Health Sciences, University of Buea, Buea, Cameroon; d Centre for Drugs and Diagnostics, Department of Tropical Disease Biology, Liverpool School of Tropical Medicine, Pembroke Place, Liverpool, L3 5QA, UK; e Department of Zoology and Animal Physiology, Faculty of Science, University of Buea, Buea, Cameroon; f Institute of Medical Microbiology, Immunology and Parasitology, University Hospital Bonn, Germany; g German Center for Infection Research (DZIF), Bonn - Cologne Partner Site, Bonn, Germany

**Keywords:** Microfilaridermia, Macrofilaricide, Onchocerciasis, *O. ochengi*, *O. volvulus*, Murine

## Abstract

**Background:**

Current treatment options for onchocerciasis are sub-optimal, prompting research and development of a safe cure (macrofilaricide). *Onchocerca ochengi,* a parasite of cattle, is used as a close surrogate for the human parasite *O. volvulus* in a murine model for pre-clinical screening of macrofilaricides. Skin from naturally infected cattle have been used in previous studies as a reliable source of parasite material. However, there is limited knowledge on how source-related factors such as the microfilaridermia status of the cattle, the nodule load and nodular worm viability may affect survival of male *O. ochengi* worms implanted in the rodent hosts. Such relationships were investigated in this study.

**Methods:**

Dermal tissue and nodules were obtained from Gudali cattle, dissected and cultured to obtain migrating microfilariae (mf) and male worms. Emerged male worms were implanted into SCID mice and Gerbils (*Meriones unguiculatus*) and recovery rates were determined upon 42 days post implantation. Finally, nodules were processed for histology and embryogram analyses to assess the nodular worm viability and fertility, respectively.

**Results:**

Of the 69 cattle sampled, 24 (34.8%) were mf^+^ and 45 (65.2%) were mf^–^. The mean nodule loads were 180.5 ± 117.7 (mf+) and 110.6 ± 102.7 (mf-) (*p* = *0.0186).* The mean male worm harvest from nodules were 76.8 ± 120.3 and 47.2 ± 33.4 (*p* = *0.2488)* for mf^+^ and mf^–^ cattle, respectively. The number of male worms per 100 nodules were 57/100 and 46/100 nodules for mf^+^ and mf^–^ cows, respectively. Female worms from nodules of mf^–^ cows had higher counts of both normal and abnormal embryos with higher proportions of dead nodular worms evinced by histology compared to those from mf^+^ cows. A total of 651 worms were implanted into mice and gerbils, out of which 129 (19.81%) were recovered. Logistic regression analysis indicated that the microfilaridermia status of the cattle (presence of mf) (OR = 4.3319; *P* = 0.001) is the single most important predictor of the success of male worm recovery after implantation into rodents.

**Conclusion:**

Microfilaridermic cattle provide a promising source of adult *O. ochengi.* Male worms from this group of cattle have a better success rate of survival in a murine implant model. Nevertheless, in the programmatic point of view, amicrofilaridermic Gudali cattle would still constitute an important source of *O. ochengi* male worms with relatively good viability after implantation into rodents.

## Highlights

• There is a need of developing safe macrofilaricide for human onchocerciasis using cattle *Onchocerca ochengi.*
• Skin from naturally infected cattle have been used as a reliable parasite source for development of murine pre-clinical screening models.
• The role of source-related factors on *O. ochengi* production and the success of pre-clinical screening models is assessed.
• Worms from positive microfilaridermic cattle are successfully recovered after their implantation into rodents.

## Graphical abstract

**Figure:**
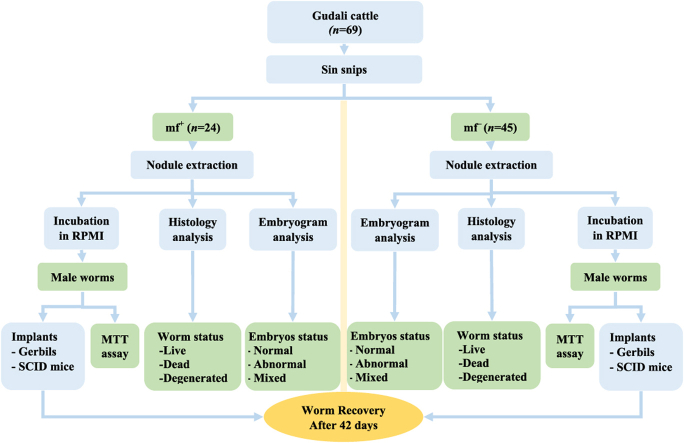

## 1 Background

Human Onchocerciasis commonly called river blindness, caused by the filarial nematode *Onchocerca volvulus,* is a debilitating parasitic disease, affecting human populations in Sub-Saharan Africa, Latin America and Yemen (Mahdy et al., 2018). Approximately 38 million people are affected with about 205 million people at risk of infection in Africa (WHO, 2018). By 2016, four countries in Latin America had been verified by WHO as having interrupted the transmission of onchocerciasis; these include Cuba in 2013, Ecuador in 2014, Mexico in 2015 and Guatemala in 2016 (Sauerbrey et al., 2018). Although there is evidence of elimination of the disease in some endemic foci in Africa, such as in Mali and Senegal, (Diawara et al., 2009; Traore et al., 2012), there is a consensus that Onchocerciasis cannot be eliminated from Africa in the near future, since control measures rely solely on Mass Drug Administration (MDA) of ivermectin, a microfilaricidal drug (Dadzie et al., 2003) with embryostatic effect (Basáñez et al., 2008). MDA is further hampered by the occurrence of severe adverse reactions to ivermectin treatment in people heavily infected with the filarial parasite *Loa loa* in areas where loiasis and onchocerciasis are co-endemic (Gardon et al., 1997). In addition, evidence of sub optimal response to ivermectin in a population of worms in some Ghanaian communities has raised concerns about the possible emergence of drug resistance by the parasite in the future (Churcher et al., 2009). Therefore, the need for a macrofilaricidal compound to overcome the present challenges and to supplement current control strategies is of great interest and several of these drugs are currently in the pipeline for screening (Hopkins et al., 2005).

Traditionally, the use of cattle naturally infected with bovine *Onchocerca*; *O. dukei, O. gutterosa, O. ochengi,* and *O. lienalis*, has been exploited as a pre-clinical system to screen potential *Onchocerca-*specific drugs with macrofilaricidal activity. *Onchocerca ochengi*, is so far considered the best model for drug screening, as a proxy for the human parasite *O. volvulus,* since it is the only *Onchocerca* species that share the same vector (*Simulium damnosum*) with *O. volvulus*. They are phylogenetically related and are both nodule forming nematodes (Trees, 1992). Techniques such as embryogram involving digestion of nodules with collagenase (Schulz-Key, 1988) for worm fecundity assessment, and histology for gross morphological assessment of nodular worm viabilities (Striebel, 1988) are extensively used for evaluating drug efficacy on *Onchocerca* species in bovine. It is however difficult to experiment on parasitized cattle since throughput is severely restricted by the logistical constrains. A potential solution to these challenges facing pre-clinical onchocerciasis macrofilaricide evaluations, is the development of a small murine model for screening potential macrofilaricidal drugs. Halliday and colleagues*,* in 2014 successfully developed a pan-filarial murine model, as pre-clinical screening system for macrofilaricide against onchocerciasis and lymphatic filariasis. In this system, *O. ochengi* whole nodules as well as adult male worms from nodules of naturally infected Gudali cattle were successfully implanted intra-peritoneally into CB.17 SCID and Gerbils (*Meriones unguiculatus*). This murine model is robust, with high throughput and is preferred over cattle because of ease of handling.

Skin from naturally infected cattle, offers a readily available and abundant supply of parasitic material in terms of mf and nodules containing adult male and female worms. Male *Onchocerca* worms are preferably used for both *in vivo* and *in vitro* drug screening since their relatively small size makes them more motile and they can freely migrate out of the nodules undamaged compared to their female counterparts, which are longer and are entangled in the nodules. Experience over several months evaluation period indicated that the prevalence of infected female cattle being moved for slaughter in the South West Region, was typically between 5 and 10%, with around 10–20 cattle being processed daily at a local abattoir, and there was frequent availability of infected cattle tissues. This parasite material, has been exploited for research purposes, for several years, most often without adequate knowledge on source related factors such as the microfilaridermic status of the cattle, their nodule load and the nodular worm viabilities, the age and the treatment histories of the cattle. These factors, may account for variations in worm quantity and quality with a consequential effect on their survival when harvested and implanted in to the murine model for drug screening. This may cause increased variation in drug screening readouts and potentially erroneous interpretations of the efficacy of drugs tested *in vivo* or in culture. This study was therefore aimed at determining initial parasitological measures from isolated *O. ochengi* predictive of long-term survival of male *O. ochengi* worms in a murine host.

## 2 Methods

### 2.1 Ethical considerations

All animals used for the experiments were infected in the laboratory of REFOTDE. All animal procedures and protocols received ethical approval by the REFOTDE (research foundation in tropical diseases and the environment) Institutional Animal Ethics Committee (RIAEC) and undertaken in accordance with UK animal regulatory standards. The study design and protocols were approved under ethical clearance 001/RIAEC/2015. Animal experimentation were in strict accordance with the international guidelines of rearing animals and use in medical research, the Animal Welfare Legislation and Policies and complied with the Animals (Scientific Procedures) Act 1986 (ASPA) and its associated codes of practice on animal housing and care (Hollands, 1986). Previous works that used the same procedures are found here (Halliday et al., 2014; Pionnier et al., 2019).

### 2.2 Extraction of nodules from cow skin

Fresh cow skins were obtained from four slaughter houses in the Southwest Region of Cameroon. The cattle were of the Gudali breed (*Bos indicus)* from the Adamawa region. Whole skin were collected after careful palpation to check for the presence of nodules, in the ventral umbilical region, the predilection site for *O. ochengi* (Wahl et al., 1994a). Upon arrival at the laboratory, the skin samples were thoroughly washed, bath in 70% ethanol and allow to dry. Individual nodules were carefully extirpated using a scalpel and forceps and place in *Petri* dishes containing RPMI-1640 medium (Sigma-Aldrich, St Louis, UK) + PSN antibiotic (20 mL/L of culture medium). From each batch of nodules extracted, a minimum of two nodules each, were fixed in 10% buffered formalin for histology. A minimum of two nodules were frozen at −80 °C in a 15 mL falcon tubes (falcon, USA), containing 5 mL of RPMI-1640 medium, for subsequent collagenase digestion for embryogram analysis. The remaining nodules were then incubated to harvest male worms. The number of nodules incubated were recorded for each cow sampled.

### 2.3 Harvesting of male O. ochengi worms from nodules

Nodules were carefully dissected with a scalpel, to avoid damaging the worms and their content squeezed out and incubated in Petri-dishes (30 nodules per *Petri* dish), containing RPMI-1640 + PSN antibiotic (Gibco life technologies, USA), at 37 °C, 5% CO_2_, for 4 h to enable male worms to migrate out of the nodules into the medium. Free, intact and motile adult males were confirmed by visualization of posterior anatomy under a dissecting microscope (Leica microsystems, Singapore) at ×1.5 magnification. The undamaged male worms were then washed twice with fresh culture medium, supplemented with 5% Fetal Bovine Serum (FBS) for implantation into the rodents (Halliday et al., 2014).

### 2.4 Microfilariae isolation from cow skin tissue and enumeration

After extirpation of nodules from the skin tissues, portions of 20 cm^2^ (5 cm × 4 cm), of the upper epidermal layer of the skin were then measured and cut out using a scalpel and incubated in *Petri-*dishes containing RPMI medium + PSN antibiotic, at 37 °C, 5% CO_2_ for 4 h. This is to enable the mf to migrate out of the dermal tissue into the medium. The medium containing the emerged mf was then passed through a fine gauze into 15 mL centrifuge tubes (falcon, USA) in aliquots of 10 mL and centrifuged (Humax 14 k human, Germany) at 2000 rpm for 10 min for the mf to settle. For mf counts, the supernatant was reduced to 5 mL, thoroughly mixed and four drops of the suspension, 10 μL each, were placed on a clean slide and the mf counted under an inverted light microscope at 40× objective (Motic). Dilution or further concentration were performed whenever the mf load was too high or too low respectively. The average number of mf thus obtained was then used to compute the total number of mfs in the 5 mL of suspension. The cattle were then grouped as microfilaraemic or amicrofilaraemic, based on the presence or absence of dermal mf.

### 2.5 Assessment of worm viability

Prior to implantation into the rodents, the viability of male worms was assessed by subjecting a few worms (2–3 worms), from each batch of worms harvested, to MTT assay (MTT, is a yellow tetrazolium bromide salt; Sigma Aldrich, USA). Briefly, single intact worms are placed in each well of a 96–well plate containing 200 μL of a solution consisting of 5 μL MTT solution (0.5 mg/mL concentration), then incubated at 37 °C, 5% CO_2_ for 2 h. After incubation, if the worms are alive they will be colored dark blue because the MTT is reduced to formazan (a dark blue precipitate that is soluble in polar organic solvents) by dehydrogenase enzymes in the cells of live worms. The stained worms are then washed twice with 200 μL of PBS (Phosphate Buffered Saline) and 200 μL of DMSO (Dimethyl Sulfoxide; Sigma Aldrich, France) added to the wells and incubated again at 37 °C for 1 h, to dissolve and release the blue formazan product. The plate was gently agitated by placing it on an agitator (Shepreth, England) to disperse and homogenize the color, and the optical density (OD) read at 490 nm in an ELISA plate reader (HumaReader, HS Human, Germany), using DMSO as the blank (Loveland et al., 1992).

### 2.6 Implantation of adult male worms into rodents

Five week-old CB.17 SCID mice and ten weeks old gerbils (*Meriones unguiculatus*) obtained from Charles River laboratory in UK and Italy respectively were maintained in the laboratory uder appropriated conditions. Conventional mice housings were used for gerbils while SCID mice were maintained in individually ventilated cages (IVCs) in the facilities of the Research Foundation in Tropical Diseases and the Environment (REFOTDE), Buea, Cameroon. The animals were fed daily *ad libitum*, while cages were cleaned and the bedding changed twice a week. For *Onchocerca* adult implants, rodents were placed under surgical anesthesia using intra peritoneal administration of a combination of ketamine and medetomidine (an anesthetic and sedative respectively), based on the animals' weights. Using a sterile scalpel, a small incision was made in the skin, around the abdominal cavity wall in the upper right quadrant. Groups of 10–23 *O. ochengi* male worms were then implanted into the animal's peritoneal cavity; 10–15 male worms were implanted into C.B17 SCID mice and 20–23 were implanted into gerbils. The skin was sutured and the animals were then administered an anti-sedative, 30 min post implantation, to enable them recover from the anesthetic and penicillin antibiotic was administered to stop any infection due to surgery. The animals were individually housed and closely monitored after recovery from anesthetic effect, till the recovery period of 42 days, the maximum period for which the worms could survive in these rodents (Halliday et al., 2014).

### 2.7 Recovery of male worms from small rodents

After 42 days post implantation, the rodents were euthanized by exposing them to a gradual increase in concentration of CO_2,_ dissected and worms were recovered by peritoneal lavage. For gerbils, 25 mL of RPMI-1640 medium plus PSN antibiotics, while for SCID mice 15 mL RPMI-1640 medium plus PSN antibiotics, were gradually introduced into the peritoneum of the animals using a glass Pasteur pipette and internal organs were gently macerated to liberate any trapped worms. The peritoneal contents were then transferred into Petri dishes containing medium to increase the chances of parasite recovery. The dishes were observed under a dissecting microscope (Leica), for worm identification and isolation. The worms recovered were counted and recorded for each animal on animal dissection and worm recovery sheets (Halliday et al., 2014).

### 2.8 Female worm extraction from nodules and fecundity assessment by of embryogram

Nodule frozen at −80 °C in RPMI-1640 following extraction, were processed for embryogram studies. The nodules were allowed to thaw at room temperature for 30–40 min cleaned off excess tissue, weighed and incubated in 15 mL falcon tubes, containing 5 mL of filtered collagenase solution (2.25 mg/mL; Sigma, USA) (Schulz-Key et al., 1980). The digested nodules were then transferred singly into Petri-dishes containing RPMI-1640 medium under a dissecting microscope. The digested nodular capsules were teased out and the female worms flushed out with RPMI-1640 medium using a dropper. Each female worm extracted was placed on a clean slide and chopped into small pieces with a scalpel into a paste-like consistency which was then washed with 1 mL RPMI-1640 medium into a mortar, placed on a 3 cm thick sponge, to absorb any shock during crushing. The contents were then crushed using a small pestle, into a fine homogenate, by applying gentle pressure to squeeze the embryos out of the female worms’ uteri without damaging them. A volume of 25 μL of the embryonic suspension was used to prepare smears which were then allow to dry overnight at room temperature, for staining. The slides were fixed with absolute methanol for 1 min, then stained with 10% Giemsa for 45 min and then read under the microscope at x 10 and confirmed at ×40 magnification. All embryonic stages both normal and abnormal were identified, counted with the help of a whole blood tally counter and classified as: normal (viable) and abnormal (degenerated) oocytes, normal and abnormal morulae, normal and abnormal coiled mf and normal and abnormal stretched mf (Schulz-Key et al., 1980).

### 2.9 Nodule processing and nodular worm viability assessment by histology

Nodule fixed in 10% formalin following nodule extraction were processed manually, following the histopathology protocol from the Michigan State University, Department of Pathology/Histology; Issued by A. Porter, HT (ASCP) C and approved by William S. Spielman. Briefly, nodules were dehydrated by incubated in ascending graded strength of alcohol (60%, 75%, 80%, 96%) followed by clearing in 100% xylene then paraffin infiltration. The paraffin blocks containing nodules, were then cut in to 5 μm sections, using a hand held semi-automated micron rotary HM 325 (Thermo Fisher Scientific, Shangai, China). The tissues sections were then stained with hematoxylin and eosin (H&E) and the slides were mounted and observed under a light microscope (Human scope) using ×10 and ×40 objectives, for confirmation of specific morphological features of the worms, to appreciate live, dead and degenerated worms using a descriptive referenced by Büttner et al. (1988).

### 2.10 Data processing and statistical analysis

Date was entered in template designed in Excel (2013), and exported to SPSS v.20 (IBM, Armonk, NY, USA) for statistical analysis and GraphPad Prism version 8 was used for plotting of graphs. The data was first checked for normality using the D'Agastino and Pearson omnibus Shapiro-Wilk normality test in GraphPad Prism version 8 software. Following success for both tests, two-tailed parametric Student's t-test was used, otherwise two-tailed Mann-Whitney *U* test was an alternative test for comparison of their distributions. The histological assessment of nodules was done based on four point scoring approach, with a score range of 0–3: 0 - Nodule without worms; nodular tissue only without worm or relic of worm; 1 - Nodules with Live worm, intact morphological structures, with embryos (productive) or without embryos (non-productive); 2 - Nodules with dead worm, abnormal features (lack or organization and degeneration), may be productive with embryonic degeneration or non-productive, and 3 - Nodules with degenerated or calcified worm, severe or advanced degeneracy, complete loss of features and presence of massive eosinophilic deposit (Splendore Hoeppli deposit).

Pearson Chi-Square test was used in SPSS to compare proportions of live, dead and degenerated nodular worms of nodular worms. Factors likely to promote male worm recovery 42 days post implantation (predictors), were determined using bivariate and multivariate logistic regression model. Six predictors were used in these analyses as described. The microfilaridermia status of the cattle as source of male worms was grouped into two categories, presence of mf (microfilaridermic) and absence of mf (amicrofilaridermic group, reference category). The mf load of the cattle, MTT scores of male worms, and number of male worms per nodule were treated in the linear scale. The histology status of nodules extracted from the cattle, were categorised based on the histological presentation of their worms as follows: 0– Nodules with live worms only (reference category); 1– Nodules with dead worms only; 2– Nodules with degenerated worms only; 3–Nodules with both live and dead worms; 4– Nodules with both live and degenerated worms; 5–Nodules with both dead and degenerated worms; 6– Nodules with live, dead and degenerated worms. The embryogram status of female worms from the nodules extracted from the cattle was scored based following a three-point score: 1–Productive (nodules with female worms having all normal embryos, reference category); 2– Non-productive (nodules with females having either empty uteri or whose embryos are all abnormal); 3–Mixed (nodules with females having a mixture of normal and abnormal embryos). Factors that significantly promoted male worm survival were determined by odd ration (OR) > 1 and those that do not promote worm survival have OR < 1. Variables that were significant in bivariate analysis, were then selected and added to multivariate. Significant differences were reported for p-values below 5%

## 3 Results

### 3.1 Nodule extraction and harvesting of male worms

The summary statistics of nodules and worms harvested from the cattle is indicated in Table 1. Skin samples were collected from 69 cattle, of which 24 (35%) were microfilaridermic (mf^+^) and 45 (65%) were amicrofilaridermic (mf^–^). A total of 207,399 microfilariae were collected and the mean mf load per surface area of the skin was 432.5 mf/cm^2^.

**Table 1:** Nodule extraction and harvesting of O. ochengi male worms from nodule mf+ and mf– cattle.

Parameter	mf^+^ Cattle	mf^–^ Cattle	Total	Unpaired *t*-test *p-* value
Cattle sampled *n* (%)	24 (35%)	45 (65%)	69	–
Nodules extracted *n*	4333	4865	9198	–
Mean ± SD nodule/cow	180.5 ± 117.7	110.6 ± 102.7	–	0.0186
Male worms harvested *n*	2457	2261	4718	–
Mean ± SD worm/nodule	76.79 ± 120.3	47.22 ± 33.37	–	0.2488
Male worm per 100 nodules	57.0	46.4	–	*–*
Mean ± SD mf count	–	8739.3 ± 17382.9	–	–

Abbreviation. mf^+^: Microfilaridermic cattle; mf^–^: Amicrofilaridermic cattle; SD: standard deviation.

**Fig. 1:**
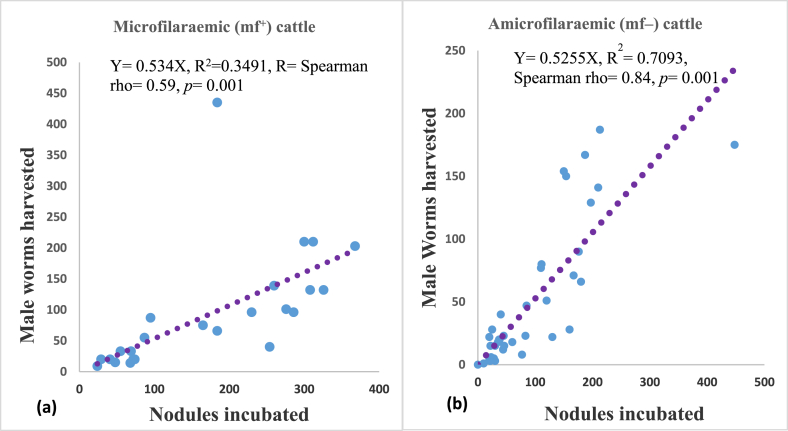
Correlation between the number of nodules incubated and the number of worms harvested from microfilaridermic cattle (a) and amicrofilaridermic cattle (b). There is a strong correlation between nodule and the male worms harvested in both groups of cattle (*p* = 0.001). The correlation is stronger for mf^–^ cattle than for mf^+^ cattle. (Spearman correction coefficient rho = 0.84 and 0.59) respectively.

A total of 9198 nodules were extracted from the 69 cow skin samples. Of these 4333 were from mf^+^ cattle, and the male worm yield of 2457, giving a nodule to male worm ratio of 1/0.57 male or 57 male worms/100 nodules incubated. A total of 4865 nodules were extracted from mf^–^ cattle, yielding 2261 male worm giving a nodule to male worm ration of 1/0.46 male or 46.4 worms per 100 nodules incubated. The mean nodule loads were 180.5 ± 117.7 and 110.6 ± 102.7 (*p* = 0.0186*)* and the mean male worm harvest from nodules were 76.79 ± 120.3 and 47.22 ± 33.37 (*p* = 0.2488*)* for mf^+^ and mf^–^ cattle, respectively (Fig. 1).

**Fig. 2:**
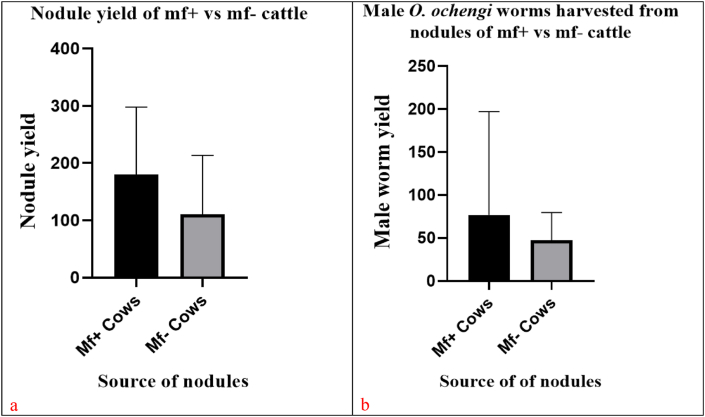
Mean nodule yields (a) and male worm harvested (b) from nodules of mf^+^ and mf^–^ cattle.

Spearman Rho correlation, shows a significant and strong correlation between nodule output and male worm yield for both microfilaridermic and amicrofilaridermic cattle (*p <* 0.001), even though it was stronger in the mf^–^ with a higher correlation coefficient of 0.84 compared to 0.59 for the mf^+^ group as indicated in (Fig. 2).

### 3.2 MTT viability and male worm recovery rates from murine host

**Fig. 3:**
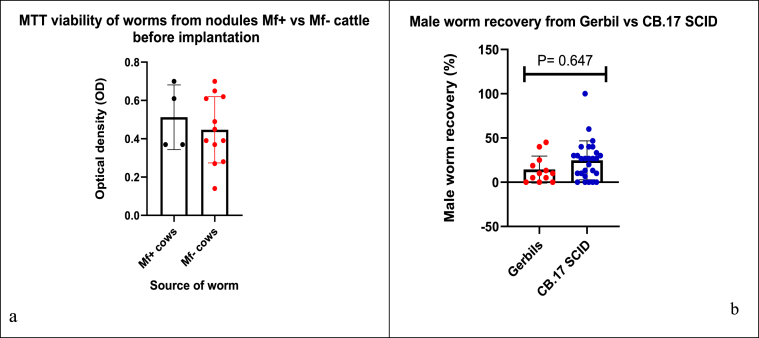
MTT viability of worms from mf ^+^ vs mf^–^ cattle prior to implantation (a); and Male worm recovery from Gerbils and CB.17 SCID mice 42 days post implantation (b). The mean ± SD of OD for worms were 0.51 ± 017 and 0.45 ± 0.17 for mf^+^ and mf^–^ cattle, *P* = 0.75. (b) Male worm recovery from Gerbils and CB.17 SCID mice 42 days post implantation, *P* = 0.647.

The mean optical density (OD) of worms before implantation where 0.51 ± 017 and 0.45 ± 0.17 for mf^+^ and mf^–^ cattle respectively, the difference was not significant according to Mann-Whiney test, *p* = 0.750 (Fig. 3a). Worms from nodules of mf^+^ cattle were more viable than those from mf^–^ cattle. A total of 39 animals, received implants of male worms, of these, 12 were gerbils and 27 were C.B17 SCID mice. Gerbils received between 20 and 23 male worms, whereas mice received 15–20 male worms averagely. For mf^+^ cows (n = 5), 75 worms were harvested from the nodules and implanted in 6/39 rodents and 24 worms were recovered 42 days post-implantation, giving a recovery rate of 32%, mean ± SD worm recovery of 30.67 ± 18.77. For mf^–^ cows (n = 1 8) 575 male worms were harvested from the nodules and implanted in 33/39 rodents, and 105 worm were recovered 42 days post-implantation, giving a recovery rate of 18.3% and a mean ± SD male worm recovery of 14.39 ± 11.80. Unpaired *t-*test showed a significant difference in the mean male recovery rate, *P* = *0.0404.* There was no significant difference in recovery between C.B 17 SCID and Gerbils, *p* = 0.647 (Fig. 3b). 2 of 27 SCID had a recovery rate of >50% whereas no Gerbil had recovery ≥50.

### 3.3 Embryogram analysis

**Fig. 4:**
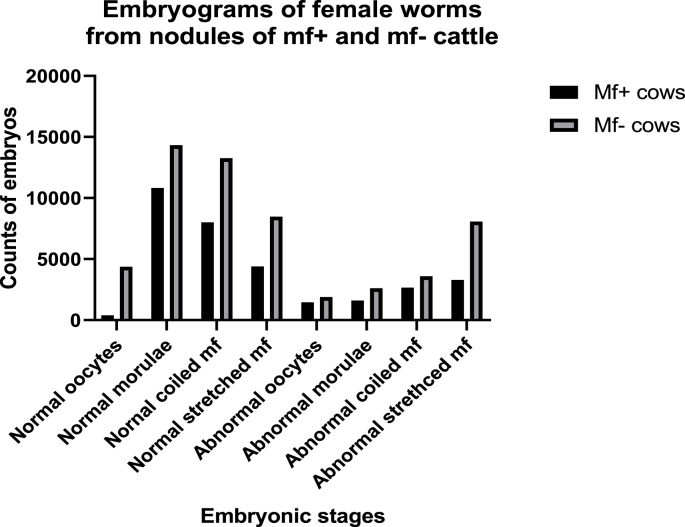
Embryogram of female worms from nodules of mf^+^ and mf^–^ cattle.

A total of 169 nodules were digested for embryogram analysis. Of these 67 were nodules obtained from mf^+^ cattle and 102 from mf^–^ cattle. A total of 177 female worms were obtained from the 169 nodules that were digested, 72 of which, were from nodules of mf^+^ cattle and 105 were from mf^–^ cattle. A total of 105 male worms were extracted from the digested nodules, 33 from nodules of mf^+^ cattle and 72 from mf^–^ cattle (Table 2). The male to female worm ratios were 1/2.2 and 1/1.5 for mf^+^ and mf^–^ cattle respectively. This indicates that, nodules from mf^+^ cattle harbor twice as many females as male worms. The differences in the mean numbers of all embryonic stages, both normal and abnormal, for mf + and mf^–^ cattle were was not significant means except for stretched mf *p* = 0.019 (Table 3). The morula was the most abundant normal embryonic stage in both the mf^+^ and mf^–^ group, meanwhile the stretched mf was the most abundant abnormal embryonic stage in the mf^+^ group (Fig. 4). The proportion of female worms with normal embryonic stages were all higher for the mf^+^ cattle than that of the mf^–^ group, even though the differences were not significant. Mf^+^ cattle, had a significantly higher proportion of non-productive females than mf^+^ cattle p< 0.001. The former group had a higher proportion of productive (fecund) female worms of 38.9%, compared with that of the latter group, 30.5%, although the difference was not significant. Productive female worms are those with an active embryogram; with all normal or predominantly normal embryos. Non-productive female worms either have all degenerated embryos or have empty uteri.

**Table 2:** Summary of embryogram of nodules from mf+ and mf– cattle.

Parameter	mf^+^ Cattle	mf^–^ cattle	Total
Nodules digested	67	102	169
Female worms recovered	72	105	177
Male worms recovered	33	72	105
Male/female ratio	1/2.2	1/1.4	–

**Table 3:** Proportion of female worms from nodule of mf + and mf– cattle with normal an abnormal embryos from embryogram.

Parameters	mf^+^ cattle n (%)	mf^–^ cattle n (%)	Chi- Square test: *p-*value
Normal embryos			
Oocytes	07 (9.7%)	24 (22.9%)	0.024
Morulae	35 (48.6%)	42 (40.8%)	0.254
Coiled mf	32 (44.2%)	35 (33.3%)	0.134
Stretched mf	34 (47.2%)	47 (44.8%)	0.747
Abnormal embryos			
Oocytes	06 (22.2%)	11 (10.5%)	0.033
Morulae	25 (34.7%)	29 (27.6%)	0.313
Coiled mf	27 (37.5%)	32 (30.5%)	0.330
Stretched mf	26 (36.1%)	37 (35.2%)	0.905

Mf^+^ = Microfilaridermic cattle, mf^–^ = Amicrofilaridermic cattle.

### 3.4 Qualitative assessment of nodular worms by histology

A total of 198 nodules were processed for histochemical analysis. Of these, 144 were obtained from mf^–^ cattle and 54 from mf^+^ cattle. Live, dead and degenerated worms as well as the reproductive status of the worms were evaluated using a modified form from Büttner et al. (1988) (additional file 1). Very few male worms were identified in sections, and their proportions were not relevant for comparison in this study. Nodules from mf^+^ cattle, had a higher proportion of live worms than those from mf^–^ cattle; 55.6% and 49.3% respectively (Table 4), even though the difference is not significant (*p* = 0.891). Conversely, nodules from mf^–^ cattle, had a higher proportion of dead and degenerated worms compared to those from mf^+^ cattle, albeit the difference was not significant. Worms from nodules of mf^+^ cattle, had significantly higher proportions of female worms with all normal embryonic stages, *p* = 0.027, 0.008, 0.035, for morula, coiled and stretched mf respectively, compared to those from mf^–^ cattle, except for oocytes *p* = 0.650 (Table 5). Very few male worms were identified in the nodule sections; hence their proportions could not be determined. This could be due to the fact that male worms are usually entangled within the female in a mating position in the nodules and so for a better appreciation of the male worms a large number of sections needed to be produced.

**Table 4:** Nodular status of worms from nodules of mf+ and mf– cattle from histology.

Histological status of worms	mf^+^ Cow n (%)	mf^–^ Cow n (%)	Chi-square test	
			*χ*2	*p-*value
Live	30 (55.6%)	71 (49.3%)	0.626	0.891
Dead	5 (9.3%)	16 (11.1%)		
Degenerated	13 (24.1%)	39 (27.1%)

**Table 5:** Proportions of fecund female worms from nodules of mf^+^ and mf^–^ cattle from Histology analysis.

Embryonic stage	mf^+^ cattle n (%)	mf^–^ cattle n (%)	Chi-square test	
			χ (Büttner et al., 1988)	*p-*value
Oocytes				
Normal	5 (9.3%)	14 (9.7%)	0.861	0.650
Abnormal	4 (7.4%)	6 (4.2%)		
No oocytes	45 (83.3%)	124 (86.1%)		
Morulae				
Normal	15 (27.8%)	20 (13.9%)	7.228	0.027
Abnormal	4 (7.4%)	5 (3.5%)		
No morula	35 (64.8%)	119 (82.6%)		
Coiled mf				
Normal	14 (25.9%)	15 (10.4%)	9.625	0.008
Abnormal	4 (7.4%)	5 (3.5%)		
No coiled mf	36 (66.7%)	124 (86.1%)		
Stretched mf				
Normal	16 (29.6%)	23 (16.0%)	6.697	0.035
Abnormal	5 (9.3%)	7 (4.9%)		
Nostretched mf	33 (61.1%)	14 (79.2%)		

### 3.5 Bivariate and multivariate regression analysis

Considering single effects on male worm survival, bivariate analysis (Table 6) indicated that, the presence of mf in the skin of the cattle (OR = 2.24, *p* = 0.0026), nodule load (OR = 0.9961, *p* = 0.0005), the number of male worms per 10 nodules (OR = 1.1762, *p* = 0.0001), and the viability of worms determined by MTT prior to implant (OR = 2.23; *p* = 0.19) were important predictors of survival of male worms. When subjected to a multivariate analysis for multiple effects on worm survival (Table 7), the presence of mf in the skin (RO = 4.3; *p* < 0.001) was the single most single most important predictor of the survival of male worms in the murine model. Male worms harvested from the nodules of cows with mf (microfilaridermic cows) have a 4 times odds of survival then worms from amicrofilaridermic counterparts. The cattle nodule load (OR = 0.9933, *p* = 0.013), the embryogram and the histology status of the worms in the nodules of the cattle are not important factors predicting the survival of worms in the murine model.

**Table 6:** Summary of bivariate analysis of the main effects of various variables on male worm recovery.

Factors	Category	Estimate (B)	Standard Error	*z-*value	ODD ratio	Confidence Interval (CI)		*p-*value
					Exp(B)	2.50%	97.50%	
Mf count	Intercept	−1.38	0.09908	−13.923				<2.10^−16^
	n	−7.10^−6^	1.704.10^−5^	−0.434	1.0000	0.9999	1.0000	0.6640
Nodule load	Intercept	−0.4713	0.22617	−2.084	–	–	–	0.0372
	n	−0.0039	0.00111	−3.487	0.9961	0.9939	0.9982	0.0005
Number of male worms/10 nodules	Intercept	−2.3225	0.3198	−7.262				0.0000
	n	0.16231	0.04269	3.802	1.1762	1.0824	1.2799	0.0001
MTTat implant	Intercept	−1.7184	0.2917	−5.891	–	–		0.0000
	OD	0.8032	0.6156	1.305	2.2327	0.6690	7.4995	0.1920
Skin mf	Intercept	−1.4988	0.1079	−13.88				<2.10^−16^
	Absent (reference)							
	Present	0.8056	0.2677	3.01	2.2380	1.3085	3.7517	0.0026
Embryo-gram score	Intercept	−1.1314	0.1286	−8.799				<2 .10^−16^
	1 (reference)							
	2	−1.1373	0.6199	−1.835	0.3207	0.0754	0.9337	0.0665
	3	−0.5115	0.2048	−2.498	0.5996	0.3993	0.8923	0.0125
Histology status	Intercept	−0.6931	0.5477	−0.693	–	–	–	0.2060
	0 (reference)							
	1	−0.9808	0.5971	−1.643	0.3750	0.1198	1.3034	0.1000
	2	−15.873	479.909	−0.033	0.0000	0.0000	0.6582	0.9740
	3	−0.9985	0.685	−1.458	0.3684	0.0955	1.4678	0.1450
	4	−0.3185	0.6207	−0.513	0.7272	0.2210	2.6282	0.6080
	5	−0.437	0.5705	−0.766	0.6460	0.2189	2.1520	0.4440
	6	−0.5921	0.631	−1.808	0.5532	0.1639	2.0306	0.3480
	7	−1.5041	1.1879	−4.555	0.2222	0.0105	1.7371	0.2050

**Table 7:** Summary of Multivariate analysis of the main effects of various variables on male worm recovery.

Factors	Category	Estimate (B)	Standard Error	z-value	ODD ratio	Confidence interval (CI)		*p-*value
					Exp(B)	2.50%	97.50%	
(Intercept)		−1.786	0.6373	−2.803	–	–	–	0.0051
Nodule		−0.003358	0.00135	−2.484	0.9966	0.9939	0.9992	0.0130
extracted								
mf count		−1.92.10^−05^	2.10^−5^	−0.949	1.0000	0.9999	1.0000	0.3426
Worms/		0.1705	0.06212	2.745	1.1859	1.0532	1.3447	0.0061
10. nodules								
Skin mf	Absent (reference)							
	Present	1.466	0.3535	4.148	4.3319	2.1705	8.7258	3.36.10^−05^
Embryogram score	1 (reference)							
	2	−0.7344	0.6614	−1.11	0.4798	0.1066	1.5526	0.2669
	3	−0.3245	0.2717	−1.194	0.7229	0.4248	1.2361	0.2323

## 4 Discussion

Cattle of the Gudali breed originating from the Adamawa region of Northern Cameroon, a commercial source of meat products, offers an abundant and relatively convenient sampling source of *O. ochengi*. Experience over a three-month evaluation period in 2014, indicated that the prevalence of infected female cattle being moved for slaughter in the South West Region was typically between 5 and 10% and that, with around 10–20 cattle being processed daily at a local slaughter houses, hence there was frequent availability of infected cow skin (unpublished data). This provides and adequate supply line of *O. ochengi* for both *in-vitro* and *in-vivo* studies. The first experiment on naturally infected cattle conducted to examine the relation between nodule load and microfilarial density in animals of different ages, demonstrated that immunity to microfilariae, but not adult worms, develops in older animals with old nodules harboring old, less reproductively viable and degenerating worms (Trees, 1992). It has been suggested that this variation in skin mf load is unlikely due to variations in the nodule load, but more likely as a result of seasonal transmission of *Onchocerca* parasite, as observed with *O. volvulus* in man (Fuglsang et al., 1976), and in *O. gutturosa* and *O. lienalis* in European cattle (Eichler, 1973). During the hot dry season, transmission of *O. ochengi* is highest, whereas in the rainy season, transmission is lowest (Wahl et al., 1994b). Wahl and colleagues in 1994 showed that *O. ochengi* adults and mf were less frequent in young Gudali cattle, peaked at the age of 4–7 years and declined in cattle older than 7 years, in both the Gudali; predominant in the Adamawa highlands of the (Guinea savanna) and the Fulani breeds; predominant in the Northern Sudan Savanna. The age-related differences in prevalence between species indicated that whenever cattle are exposed to high rate of *O. Ochengi* transmission, high prevalence of infection are reached in the middle-aged animals (4–7 years), and declines in older animals (above 7 years). Such a decline in prevalence with age in hyper-endemic areas indicates a naturally acquired resistance against *O. Ochengi* mf in old cattle. Also, it has been shown with *O. volvulus* that, in areas of the former Onchocerciasis Control Programme in West Africa, a sustained decrease in transmission brings about an ageing of the worm population (Karam et al., 1987), associated with an increase in the proportion of old female worms harboring degenerating stretched mf (Schulz-Key and Karam, 1986). Wahl and colleagues, noticed an increase in mean residual mf following collagenase digestion of skin biopsies. The slow emergence rate of mf may be due to large size of the biopsies at incubation and the tight texture of cattle skin, such that only peripheral mf received enough stimuli or were capable of migration to the outer surface of the biopsies at elevated temperatures. The sensitivity of the skin snip method could be enhanced by mincing the skin biopsies and prolonging the time of incubation to 24 h at 37 °C to allow maximum time for the mf lodged in deeper skin layer to emerge. This may account for the low (35%) mf prevalence observed in this study. A limitation to this study is the fact that the age, sex of the cattle and seasonality were not taken into account, as this would have provided some relevant information.

The development of a ‘pan-filarial’ small animal murine research model, consisting of the inbred SCID mice and the Mongolian gerbils (*Meriones unguiculatus*), an outbred laboratory rodent susceptible to a number of other filariae has enhanced macrofilaricidal drug research (Halliday et al., 2014). This model, is sufficiently robust, with adequate capacity and throughput screening of existing and future pre-clinical candidate macrofilaricides. With this, it is now possible to maintain the parasite for over 5 weeks, enough time to evaluate macrofilaricide efficacy *in-vivo*. Sources of *O. ochengi* male worm for implant were microfilaridermic and amicrofilaridermic cattle. And worms from the former group survived better than those from the latter with a significantly higher mean recovery of 30.60 ± 18.75 and 14.38 ± 11.95, *p* = 0.042 for mf^+^ and mf^–^ cattle. This could be due to the fact that these worms represent an older and more fragile population of worms with reduced viability. Embryogram analysis revealed a more productive proportion of female worms from nodules of mf^+^ cattle. Abnormal embryos were more abundant in female worms from microfilaridermic cattle than in those from the mf^–^ group (Fig. 4). This could be accounted for by the higher male to female ratio in the former than in the latter. Hence more female worms are being fertilized since males can move from one nodule to another. The advanced embryos in very productive female worms, embryonic stages such as the morulae and coiled mf are febrile and more metabolically active, hence more susceptible to degeneration when they accumulate in the females' uteri than the more advanced stretched mf (Schulz-Key et al., 1980).

**Fig. 5:**
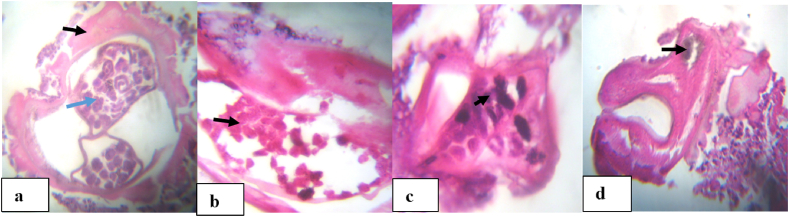
Histological sections of O. ochengi nodules with death and degenerating worms. (a) dead female worm with abnormal (degenerating) morulae (blue arrow) and a mass of eosinophilic deposit (Splendore-Hoeppli; SH deposit) on worm cuticle (Black arrow). (b–c) indicate female worms in state of degeneration with complete loss of internal organs and worm structure. Abnormal microfilariae and morulae in a state of degeneration in uterus of female worms in (b) and (c) respectively (arrows). (d) female worm in state of advanced degeneration (calcification), with distorted brownish gut (arrow) and SH deposit in the worm. (For interpretation of the references to color in this figure legend, the reader is referred to the Web version of this article.)

**Fig. 6:**
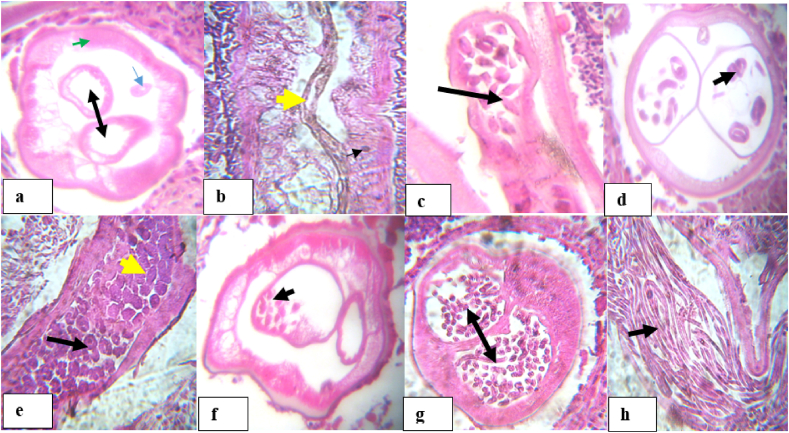
Histological sections of *O. ochengi* nodules with live and fecund female worms. (a) cross-section of female worm with empty uteri (black arrow heads), a gut (blue arrow) and muscular hypodermis (green arrow head); b: logitidinal section of worm with gut (yellow arrow) and hypodermel nucleus (black arrow heads); (c) female worm with oocytes in uterus (arrow). (d) female with coiled mf (arrow); (e) Female with pretel or horse shoe (intermidiate form between coiled mf & morulae (black arrow) and mature morulae (yellow arrow); (f–g) cross-section of female with few and abundant mf respectively; (h) Longitudinal section of female worm with nucleated mfs (arrow). (For interpretation of the references to color in this figure legend, the reader is referred to the Web version of this article.)

It is assumed that the female worms in the nodules are tightly coiled together by chance or accidently, hence a random distribution of the various parts of the body and its internal organs within the nodule can be expected. The percentage of dead and more so of calcified (degenerated) female worms, increase with increasing age of the worm population and these proportions were significantly higher in amicrofilaridermic group than in the microfilaridermic group (Fig. 5a–d). This suggests and aging non-productive worm population consistent with embryogram results. Live female worms with normal and active embryos, equally predominated in the microfilaridermic group (Fig. 6a–e), with the greatest population of stretched mf, indicating a reproductively active worm population in this group compared to the amicrofilaridermic group. Very few male worms were identified in the nodule sections; hence their proportions could not be determined in this study. This is due to the fact that male worms are usually entangled within the female in a mating position in the nodules and so for a better appreciation of the male worms a large number of sections need to be produced. The few male worm sections indicated mature fecund male worms that had already copulated or with the potential of copulating. The determination of the number of worms in a nodule is the most contentious area in nodule examination where the differences between individuals reporting on the number of worms in a nodule is significant (Duke et al., 1990). For very small nodules which contain only one worm or two worms, of which one is a male and the other a female, there is no significant disagreement between individuals in the number of worms per nodule (Renz et al., 1995). Large nodules containing multiple worms provide significant variations between individuals reporting on the number of worms (Duke et al., 1990). Sections with the same genital contents can be attributable to the same worm. Because of the fixation and processing of nodules, all worms examined by the histological technique are dead even before the examination, for this reason the terms “live” and “dead” worms in histological assessments refers to the state of the worms at the time of nodulectomy. The distinction between live and dead worms is very important for studies based on the evaluation of compounds for macrofilaricidal purposes (Büttner et al., 1988).

The microfilaridermic status of the cattle from regression analysis, is the single most reliable factor that could be used as a proxy to predict the success of the survival rate of Worms in a murine host. This is supported by a higher odd of survival in the murine host (OR = 4.33; *p <* 0.001) for the worms from nodules of microfilaridermic cows, compared to their amicrofilaraemic counterparts. This is reflected in the higher recovery rate recovery rate of 32.0% (mean ± SD; 30.60 ± 18.75) for the latter group and 18.3%; mean ± SD; 14.28 ± 11.92 (*p* = 0.042) for the former. However, the physiological conditions of the mice and other external fact factors such as physical integrity of the worms harvested from the nodules, prior to implantation, may also have an impact on the survival of the worms in this system. Other factors such as the viability of worms prior to implantation determined by MTT assay, the cattle nodule load, and the histology and embryogram status of the nodule from cattle are not strong covariates to consider in predicting the outcome, of male worms implanted in a murine model in terms of success of their survival rate.

## 5 Conclusions

Microfilaridermic cattle are promising source of parasite material in terms of mf, nodule and male worm yields. The mf status of cows is an important predictor of male worm survival in a murine model, given that worms from this group of cattle had a better recovery rate. However, amicrofilaridermic cows are not without their value and still remain a reliable source of parasite material.

## Consent for publication

Not applicable.

## Data availability

The data that support the findings of this study are available from the corresponding author upon reasonable request.

## CRediT author statement

Desmond Akumtoh: Conceptualization, Methodology, Investigation, Formal analysis, Writing - Original Draft, Abdel Jelil Njouendou: Methodology, Validation, Formal analysis, Writing - Review & Editing, Haelly M. Metuge: Investigation, Writing - Original Draft, Hanna Sjoberg: Methodology, Writing - Review & Editing, Nicolas Pionnier: Methodology, Writing - Review & Editing, Valerine Chunda: Investigation, Narcisse Victor Gandjui: Investigation, Formal analysis, Bertrand Lontum Ndzeshang: Investigation, Formal analysis, Fanny Fri Fombad: Investigation, Raphael Abong: Formal analysis, Peter Enyong: Conceptualization, Validation, Jerome Fru-Cho: Validation, Mathias Enyong Esum: Validation, Manuel Ritter: Methodology, Resources, Writing - Review & Editing, Mark Taylor: Methodology, Resources, Joseph Turner: Methodology, Resources, Writing - Review & Editing, Samuel Wanji: Conceptualization, Validation, Methodology, Writing - Original Draft, Writing - Review & Editing, Supervision.

## Source of funding

This work was supported by a Bill & Melinda Gates Foundation Grand Challenges Explorations grant (no. OPP1119043) to JDT, SW, and MJT. In addition, SW is financially supported by the Federal Ministry of Education and Research (BMBF, initiative Research Networks for Health Innovations in sub-Saharan Africa: TAKeOFF) and by the Horizon 2020 Framework Programme: HELP. MR is a member of the German Center of Infectious Disease (DZIF). The funders had no role in study design, data collection and analysis, decision to publish, or preparation of the manuscript.

## Declaration of competing interest

The authors of this work have no financial, personal or professional interests that could have been construed to influence this manuscript.

## Figures and Tables

**Additional file 1:** Histology evaluation form for nodular worm viability & fecundity assessment

Date:	Nodule N^o^:	FEMALE															MALE															
																													
STRUCTURE	FEATURE																															
GENITAL	Normal																															
	Minor damage																															
	Moderate damage																															
	Severe damage																															
HYPODEMIS	normal																															
	Vacuole/granulated																															
	swollen																															
	shrunken																															
	Abnormal nuclei																															
	Minor damage																															
	Moderate damage																															
	Severe damage																															
	Worm calcified																															
GUT	Normal with nuclei																															
	Swollen																															
	Shrunken																															
	Distorted borders																															
	Minor/moderate																															
	degenerated																															
	Severe damage																															
OOCYTES	normal																															
	Distorted border																															
	Minor damage																															
	Moderate damage																															
	Severe damage																															
MORULAE	normal																															
	Loss of organization																															
	Remnant																															
	Minor/moderate. degenerate																															
	Severe damage																															
PRETZEL (COILED MF)	Normal																															
	Vacuole/fragment																															
	disintegrated																															
	Minor/moderate degenerate																															
	Severe damage																															
MICROFILARIA	Normal																															
	Vacuole/fragment																															
	Disintegrated																															
	Minor/moderate.degenerated																															
	Mf remnant																															
REMARKS																																

## List of abbreviations

DMSO: dimethyl Sulfoxide
H&E: hematoxylin and eosin
IVM: individually ventilated cages
MDA: mass drug administration
Mf: microfilaria
MTT: (3- [4,5-dimethylthiazol-2– yl]-2,5-diphenyl tetrazolium bromide
PBS: phosphate buffered saline
PSN: penicillin, streptomycin, and neomycin
RPMI: Roswell Park Memorial Institute
SCID: severe combined immune deficiency
SD: standard deviation
SH: splendore hoeppli
UK: United Kingdom
